# Cordycepin prevents the esophageal stricture formation in the alkali-burn rat model by exerting anti-inflammatory and antifibrotic effects

**DOI:** 10.1590/ACB360302

**Published:** 2021-03-15

**Authors:** Gulcin Ercan, Yuksel Altinel, Onur Olgac Karagulle, Hakan Yiğitbaş, Nadir Adnan Hacım, Serhat Meriç, Nihat Buğdaycı, Rumeysa Ilbar Tartar, Burcu Biltekin, Erkan Yavuz, Osman Bilgin Gulcicek, Ali Solmaz, Atilla Çelik

**Affiliations:** 1 MD. University of Health Sciences – Istanbul Bagcilar Training and Research Hospital – Department of General Surgery– Istanbul, Turkey.; 2 MD, MSc. University of Health Sciences – Istanbul Bagcilar Training and Research Hospital – Department of General Surgery – Istanbul, Turkey.; 3 MD. University of Health Sciences – Istanbul Training and Research Hospital – Department of General Surgery – Istanbul, Turkey.; 4 MD. Sanliurfa Education and Research Hospital – Department of General Surgery – Sanliurfa Province Health Directorate – Sanliurfa, Turkey.; 5 MD. University of Health Sciences – Sisli Hamidiye Etfal Training and Research Hospital – Department of General Surgery – Istanbul, Turkey.; 6 PhD. Istanbul Atlas University – Faculty of Medicine – Department of Histology and Embryology – Istanbul, Turkey.; 7 MD, Associate Professor. University of Health Sciences – Istanbul Bagcilar Training and Research Hospital – Department of General Surgery – Istanbul, Turkey.; 8 MD, PhD, Associate Professor. Camlica Erdem Hospital – Department of General Surgery – Istanbul, Turkey.

**Keywords:** Alkalies, Esophageal stenosis, Fibrosis, Prednisolone, Rats

## Abstract

**Purpose:**

To investigate the efficacy of cordycepin, an adenosine analogue, on prevention of esophageal damage and stricture formation due to esophageal caustic burns in rat model comparing with prednisolone.

**Methods:**

Caustic esophageal burn was introduced by 37.5% of NaOH to distal esophagus. Thirty-two Wistar albino rats were divided in four groups: sham rats undergone laparotomy, treated with 0.9% NaCl; control rats injured with NaOH without cordycepin treatment; cordycepin group injured with NaOH, treated with 20 mg/kg cordycepin; prednisolone group injured with NaOH, treated with 1 mg/kg prednisolone for 28 days. Efficacy was assessed by histopathological and immunohistochemical analysis of esophageal tissues.

**Results:**

Cordycepin treatment significantly decreased inflammation, granulation tissue and fibrous tissue formation and prevented formation of esophageal strictures shown by histopathological damage score and stenosis indexes compared to control group (p < 0.01). These effects are relatively more substantial than prednisolone, probably based on attenuation of elevation of proinflammatory cytokines hypoxia-inducible factor 1-alpha (HIF-1?), tumor necrosis factor alpha (TNF-?), proliferative and fibrotic factor fibroblast growth factor 2 (FGF2) and angiogenic factor vascular endothelial growth factor A (VEGFA) (p < 0.05).

**Conclusions:**

The findings suggest that cordycepin has a complex multifactorial healing process in alkali-burned tissue, more successful than prednisolone in preventing the formation of esophageal strictures and may be used as a therapeutic agent in the acute phase of esophageal alkali-burn.

## Introduction

Accidental ingestion of alkalis results in severe esophageal injuries and stenosis formation^1^. Alkali ingestion causes liquefaction necrosis resulting in greater tissue penetration and potential complications. The acute phase of alkali-burn starts with the inflammatory phase where pro-inflammatory and profibrogenic cytokines exert their deleterious effects on the esophageal tissues, resulting in stricture formation and esophagus stenosis^2^. There are several treatment protocols devoted to achieving anti-inflammatory and antifibrogenic actions in caustic burns^3^^–^ 5. Among these treatments, corticosteroids can decrease inflammation, granulation tissue and fibrous tissue formation; however, when used in the treatment of caustic injuries of the esophagus, it may not have any significant benefit in preventing esophageal strictures^6^. The routine use of antibiotics in patients with caustic ingestion is also controversial^2^. The use of H2 receptor antagonist and proton pump inhibitors (PPIs) in caustic ingestion injuries seems rational and reasonable if well tolerated^7^. Therefore, there has not been a standardized method in treatment of esophageal stricture formation and fibrosis induced by alkali-burns.

Up to now, the effects of many anti-inflammatory and antioxidative agents have been investigated in preventing esophageal fibrosis and stricture formation in experimental caustic esophageal burn models^3^^–^ 5 ^,^ 8 ^,^ 9. Cordycepin, 3’-deoxyadenosine (9-3-deoxy-β-d-ribofuranosyl) adenine, is an active ingredient that was isolated from *Cordyceps militaris* and a natural derivative of adenosine, possessing multiple pharmacological activities including antioxidation, antimicrobial, anti-inflammation^10^^–^ 12. However, whether cordycepin can prevent caustic burn-induced esophageal stricture formation and stenosis and fibrosis remains unknown. In this study, the aim was to evaluate the effects of cordycepin on inflammation, stricture formation, fibrosis, histopathology and stenosis in a rat model of esophageal alkali-burn and compare the possible effects with a corticosteroid treatment, suggesting that cordycepin could be a suitable candidate for studying treatment of esophageal caustic injuries.

## Methods

This experimental study was approved by the Local Ethical Committee of the University of Health Sciences Bagcilar Training and Research Hospital (Number: 2018/10 Date: January 29, 2018). All procedures in the study were applied in accord with the Brazilian law and the Council for International Organization of Medical Sciences (CIOMS) and followed the Animal Research Reporting of in Vivo Experimental (ARRIVE) guideline.

Thirty-two Wistar albino rats weighting 250 ± 30 g and aged between 11–13 weeks were used in this study. All rats were housed in metal cages at 22 °C with a 12-hour light/dark cycle for 10 days before the study and handled according to the principles and procedures outlined in the National Institute of Health guide for the Care and Use of Laboratory Animals. The rats were starved for one night prior to the laparotomy. After the laparotomy, the animals were given free access to pellet feed and water and were housed under laboratory conditions for 28 days. The rats were divided into four groups of 7 each, as given below:

Sham group: rats in this group underwent a laparotomy without establishment of esophageal alkali-burn, then administrated with 1 mL dose of 0.9% saline injected intraperitoneally for 28 days.

Control group: rats in this group underwent a laparotomy with establishment of esophageal alkali-burn, then administrated with a daily dose of 0.9% saline injected intraperitoneally for 28 days.

Cordycepin group: rats in this group underwent a laparotomy with establishment of esophageal alkali-burn, then treated with a daily dose of 20 mg/kg cordycepin (Toronto Research Chemicals Inc, Canada) injected intraperitoneally for 28 days. The time interval between induction of caustic injury and the intraperitoneal instillation of cordycepin was 10 min, determined according to the treatment schedule of Turkey, where the median ambulance transport time was approximately 10 min^13^.

Prednisolone group: rats in this group underwent a laparotomy with establishment of esophageal alkali-burn, then treated with a single daily dose of 1 mg/kg prednisolone injected intraperitoneally for 28 days. The time interval between induction of caustic injury and treatment was 10 min.

### Esophageal alkali-burn model

Laparotomy was performed under the intraperitoneal anesthesia with 60 mg/kg ketamine and 10 mg/kg xylazine. Esophageal alkali-burn model was established according to the literature^3^. Shortly, a catheter was passed through the mouth and inserted into 1.5 cm upper segment of the abdominal esophagus. To prevent the leakage of caustic agent into the stomach, the cardioesophageal junction was tied from the outside and, to prevent the aspiration into the respiratory system, the esophagus was tied from the bottom of the proximal diaphragm. One mL solution of 37.5% NaOH (pH = 12) was infused for 90 seconds and then aspirated. Subsequently, the injured segment was irrigated with distilled water for 60 s. The proximal 2/0-suture was cut and drawn into the catheter with negative pressure. Subsequently, the distal 2/0-suture was cut and the laparotomy was closed^3^.

### Histopathological analysis

At the end of the experimental procedures, the rats were sacrificed by decapitation under anesthesia and 1.5 cm of esophageal tissue was excised for histopathological analysis. The proximal portion of the damaged segment was fixed by 10% neutral formaldehyde and stored at 4 °C until evaluation. Fixed tissue samples were prepared by routine histological technique for paraffin embedding, 5 *μ*m-thick sections from paraffin-blocks were deparaffinized and rehydrated, then stained with Masson’s trichrome and evaluated under a light microscope and were photographed (Olympus BX61, Tokyo, Japan). The histopathological tissue damage score (HDS) was used to determine the severity of injury and collagen deposition in esophagus tissue and stenosis index (SI) was used to determine the severity of esophageal stenosis in hollow organs3. For HDS, sections were scored on a scale (none, 0; mild, 1+; marked, 2+) in three categories (collagen deposition in the submucosa, damage to the muscularis mucosa and damage and collagen deposition in the tunica muscularis) for a total score of 0–5^14^. For SI, the mean esophageal wall thickness and lumen diameters were measured from four different locations and SI was calculated by histologist according to the literature^15^.

### Immunohistochemical analysis

Five *μ*m-thick sections were deparaffinized and rehydrated in graded alcohol series. Commercially available monoclonal antibodies against hypoxia-inducible factor 1-alpha (HIF-1α), tumor necrosis factor alpha (TNF-α), fibroblast growth factor 2 (FGF2) and vascular endothelial growth factor A (VEGFA) (Abcam, Cambridge, UK) were used. Histostain-Plus Broad Spectrum Kit (95-9943-B Zymed Lab. Ins. San Francisco CA, USA) was used for immunoperoxidase staining. The whole procedure was performed using a combination of microwave oven heating for antigen retrieval and standard in-direct streptavidin-biotin-peroxidase method. Endogenous peroxidase activity was blocked by hydrogen peroxide (3%). Each section was then incubated for 15 min at room temperature with blocking solution to stop cellular peroxidase activity. The sections were incubated with antibodies overnight at 4 °C and then washed with phosphate buffer saline (PBS). Specific staining was performed with the biotinylated universal secondary antibody, horseradish peroxidase-streptavidin complex, supplied by the kit. Amino-ethyl-carbazole (AEC) staining liquid kit (Sigma Aldrich, St. Louis, Missouri, USA) was used as a chromogen. Immunohistochemical staining was evaluated semiquantitatively using a modified H-SCORE analysis16 that assigned numerical values of 0–300 to the staining intensity at 10 different areas and all scores were averaged to generate a total score.

### Statistical methods

All data were expressed as mean ± standard deviation with all rats per group. GraphPad Instat statistical program (GraphPad Software, San Diego, CA, USA) was used for statistical analysis. Following the assurance of normal distribution of data, one-way analysis of variance (ANOVA) with the Tukey–Kramer post-hoc test was used for multiple comparison. If not normally distributed, Kruskal–Wallis test (nonparametric ANOVA) with Dunn’s multiple comparisons test was used for multiple comparison Values of p < 0.05, p < 0.01 and p < 0.001 were regarded as significant.

## Results

Twenty-six rats survived during the study, while two rats from the control group died due to the esophageal perforation and aspiration in the first 24 h of the procedure.

### Histopathological findings

**Figure 1:**
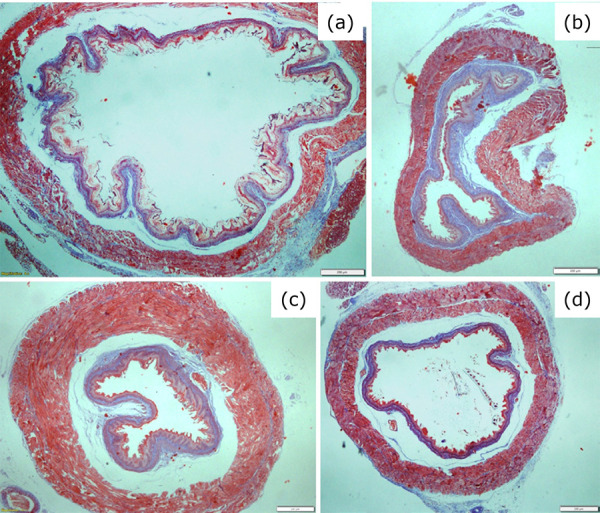
Histological images of esophageal tissues of the rats in sham group **(a)**, control group **(b)**, cordycepin group **(c)** and prednisolone group **(d)**. Magnifications × 4, Masson trichrome.

Normal histological structure of esophagus was observed in the sham group (Fig. 1a). The histological samples of the control group demonstrated an esophageal alkali-burn histopathology with a constricted lumen and increased submucosal connective tissue (Fig. 1b). The histological samples of the cordycepin-treated rats demonstrated similar characteristics to the sham group (Fig. 1c). In the prednisolone-treated group, histopathological changes were persistent and the increased submucosal connective tissue was still present (Fig. 1d).

The histopathological images and HDS data of esophageal tissues of all groups are presented in Fig. 1 and Table 1, respectively. There was a significant difference between the HDS of four groups (p = 0.0002). Histopathological tissue damage score of all rats in sham group was zero while those of the control group significantly increased compared to other groups (p < 0.0001). The HDS of rats in the cordycepin group were significantly lower compared with control group but not different from the sham group. Histopathological tissue damage score for fibrosis development was lower in cordycepin-treated rats than in prednisolone-treated rats. Histopathological tissue damage score of prednisolone group was significantly higher than the sham group (p < 0.05) but lower than the control group (Table 1).

**Table 1:** The HDS of esophageal tissues of rats.

Groups	Mean ± SD	Median [range]	P value
Sham	0 ± 0	0 [0]	0.0002
Control	4.4 ± 0.89***	5 [3–5]	
Cordycepin	1.4 ± 0.55	1 [1–2]	
Prednisolone	2.6 ± 0.98*	3 [1–4]	

* p < 0.05 and

*** p < 0.001 vs. sham group.

The SI of esophageal tissues of all groups are presented in Table 2. There was a significant difference between four groups (p = 0.0019). The lowest SI was calculated in the sham group and the highest was in the control group. Stenosis index of cordycepin group was comparable with those of sham group, but significantly lower than the control group (p < 0.05). Stenosis index of prednisolone was also comparable with those of the sham and cordycepin groups (Table 2).

**Table 2:** The SI of esophageal tissues of rats.

Groups	Mean ± SD	Median [range]	P value
Shamw	0.37 ± 0.11	0.61 [0.45–0.93]	0.0019
Control	0.62 ± 0.14**	0.40 [0.18–0.48]	
Cordycepin	0.41 ± 0.14*	0.42 [0.18–0.59]	
Prednisolone	0.55 ± 0.17	0.55 [0.30–0.84]	

* p < 0.05 vs. control group and

** p < 0.01 vs. sham group.

Totally, these findings designated that the cordycepin treatment considerably reduced the histopathological damage due to the esophageal alkali-burn, while prednisolone treatment did moderately. Also, the cordycepin treatment significantly reduced the SI better than prednisolone treatment in esophageal alkali-burn.

### Immunohistochemical findings

**Figure 2:**
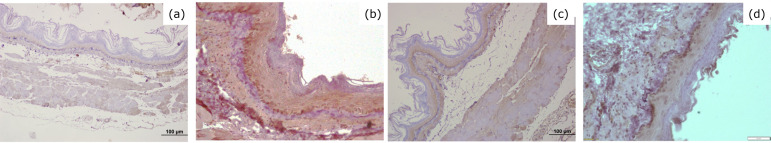
The immunohistochemical analysis of HIF-1α in sham group, × ٢٠ (**a**), control group, × ٤٠ (**b**), cordycepin group, × 20 (**c**) and prednisolone group, × 40 (**d**).

**Figure 3:**
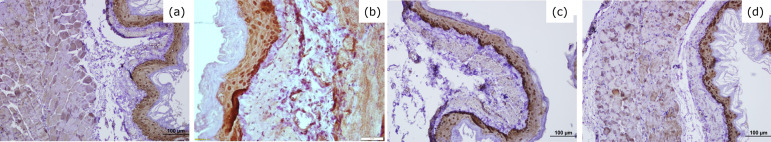
The immunohistochemical analysis of TNFα in sham group × 20 (**a**), control group, × 40 (**b**), cordycepin group, × 20 (**c**) and prednisolone group, × 20 (**d**).

Table 3 summarizes the immunohistochemical findings for HIF-1α, TNF-α, FGF2 and VEGFA detected in the esophageal tissues. As proinflammatory factors, HIF-1α and TNF-α were significantly marked by immunostaining of the esophageal tissue of esophageal alkali-burn rats, especially the basal epithelial part of the esophageal mucosa (Figs. 2 and 3). There was a very slightly positive staining for HIF-1α in sections of esophagus tissues of sham-operated rats (Table 3). In contrast, the highest HIF-1α signal was present in the control group (p < 0.001). Additionally, a significantly stronger immunostaining for TNF-α was observed in the esophageal alkali-burn (control) group. However, the cordycepin and prednisolone treatments in esophageal alkali-burn showed a significantly decreased immunostaining for HIF-1α compared with the control group (p < 0.001 and p < 0.01, respectively). Immunostaining score for TNF-α in the cordycepin group was comparable with the sham group, as well as significantly decreased compared with the control group (p < 0.05), while the score in the prednisolone group was significantly higher than the sham group (p < 0.05) (Table 3).

**Table 3:** H-scores of the immunohistochemical analysis of esophageal tissues of rats.

Groups	HIF-1α	TNF-α	FGF2	VEGFA
Sham	7.98 ± 3.33	31.23 ± 4.0	22.96 ± 9.9	29.14 ± 9.7
Control	80.39 ± 9.8***	68.08 ± 11.8***	72.33 ± 18.5***	77.31 ± 19.2***
Cordycepin	34.8 ± 20.1* ^,^ †††	30.13 ± 11.0†	24.13 ± 8.6†††	45.96 ± 19.97†
Prednisolone	45.72 ± 13.9*** ^,^ ††	50.58 ± 14.1*	46.10 ± 13.0* ^,^ †	51.95 ± 12.0
**P value**	< 0.0001	< 0.0001	< 0.0001	0.0011

Hypoxia-inducible factor 1-alpha (HIF-1α), tumor necrosis factor alpha (TNF-α), fibroblast growth factor 2 (FGF2) and vascular endothelial growth factor A (VEGFA). All data are given in mean ± standard deviation.

* p < 0.05 and

*** p < 0.001 vs. sham group.

† p < 0.05,

†† p < 0.01,

††† p < 0.001 vs. control group.

**Figure 4:**
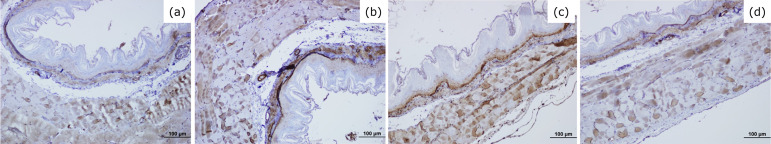
The immunohistochemical analysis of FGF2 in sham group, × 20 **(a)**, control group, × 20 **(b)**, cordycepin group, × 20 **(c)** and prednisolone group, ×20 **(d)**.

As a marker for granulation, collagen biosynthesis and cell proliferation, the FGF2 protein was detected in the esophageal alkali-burn rats, especially in the lamina propria of esophageal mucosa (Fig. 4). There was a higher positive staining for FGF2 in sections of esophagus tissues of the esophageal alkali-burn (control) group than the sham and cordycepin groups (p < 0.001) (Table 3). In contrast, the FGF2 signal was significantly decreased in the cordycepin and prednisolone group compared with the control group (p < 0.001 and p < 0.05, respectively). However, the FGF2 immunopositivity was still higher in the prednisolone-treated rats than the sham rats (p < 0.05).

**Figure 5:**
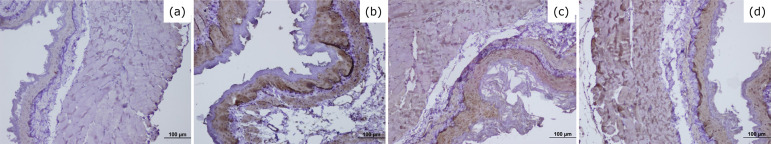
The immunohistochemical analysis of VEGFA in sham group, × 20 **(a)**, control group, × 20 **(b)**, cordycepin group, × 20 **(c)** and prednisolone group, × 20 **(d)**.

As a common marker for angiogenesis, VEGFA signal was detected in the microvessels (endothelial cells and pericytes) of the epithelium, muscularis propria, in muscularis mucosa and in large submucosal vessels of the alkali-burned esophagus (Fig. 5). Vascular endothelial growth factor A (VEGFA) signal was significantly enhanced in the control group compared with those of the sham group (p < 0.001). The cordycepin treatment significantly reduced this signal in esophagus compared with the control group (p < 0.05). However, VEGF signal was still detected higher in the prednisolone group compared with the sham group (Table 3).

## Discussion

In this esophageal alkali-burn model, the cordycepin was used, which is a known antioxidant, antimicrobial, anti-inflammatory agent given intraperitoneally. as a new treatment modality. It was demonstrated that the cordycepin treatment decreased the inflammation, granulation tissue and fibrous tissue formation and prevented the formation of esophageal strictures shown by HDS and SI calculated for the esophageal tissues. These effects of cordycepin are probably based on the attenuation of elevation of proinflammatory cytokines HIF-1α, TNF-α, the proliferative and fibrotic factor FGF2 and lastly the angiogenic factor VEGFA, suggesting a complex multifactorial healing process in the alkali-burned tissue. Another interesting finding was that the cordycepin was found to be relatively more effective than the prednisolone, one of the corticosteroids which is not very successful in preventing the formation of esophageal strictures in corrosive burns.

The cordycepin, a natural derivative of adenosine, exhibits a variety of clinical health effects including immunomodulatory, anticancer, antioxidant, anti-inflammatory and antimicrobial activities^10^^,^ 11 ^,^ 17. It was reported first that cordycepin could inhibit the biosynthesis of purine and participate in the synthesis of RNA and/or DNA^18^ or exerted its biological activity through a signal involving a reactive oxygen species (ROS) mediated caspase pathway^19^. It is confirmed that cordycepin can be converted into 5’ mono-, di- and triphosphates in vivo to inhibit the activity of enzymes and interfere a number of biochemical and molecular processes^20^. Cordycepin has been shown to attenuate age-related oxidative stress and enhance antioxidant capacity in rats^21^. It has also been shown to prevent rat hearts from ischemia/reperfusion injury partially by activating antioxidant defense^22^. Though cordycepin represents a promising agent for the potential clinical applications, its effect in any kind of medical burn, especially in the alkali-burns, was not identified before. Therefore, this is the first study showing the efficacy of cordycepin on the preventionof esophageal damage and stricture formation due to the esophageal caustic burns in a rat model.

During the early stages of caustic injury, acute necrosis and thrombosis followed by the inflammation and oxidative stress occur^23^. Collagen deposition and fibrosis followed by stricture formation by the second or third week is the natural course of the healing when left untreated^24^. Therefore, an ideal agent which can accelerate wound healing and prevent esophageal stricture formation is needed for the early treatment of a caustic esophageal injury. Therefore, the main goal of the medical treatment must be the prevention of inflammation at the burn site and also to protect the neural transmission in the deeper tissue. This effect may also contribute to the reduction of stricture formation, especially by reducing inappropriate contraction of the esophageal wall during the post-burn healing phase. To prevent stricture formation, many experimental studies have been performed to evaluate the therapeutic efficacy of agents such as beta-aminopropionitrile, platelet-rich plasma, sucralfate, trimetazidine, pentoxifylline, 3-amino-benzamide (3-AB) and ketotifen^3^^,^ 25 ^,^ 26. Also, some chemical components from a few species of mushrooms including golden needle mushroom^27^ and *Agaricus blazei* 28 were reported to possess strong anti-inflammatory activity in burned rats, suggesting a possible candidate to be a promoting agent of the burn wound healing. In the present study, cordycepin isolated from *C. militaris*, which is a species of fungus in the family Cordycipitaceae^29^, was used to investigate its therapeutic effects in the esophageal caustic burns in a rat model. The esophagus sections from rats receiving the cordycepin treatment following the induction of caustic burn revealed a more normal morphology than those of untreated rats and even those of prednisolone treated rats.

Histopathological scores, which determine the tissue damage according to collagen deposition in the submucosa, damage to the muscularis mucosa and damage and collagen deposition in the tunica muscularis, showed that the cordycepin treatment ameliorated the esophageal alkali-burn histopathology by preventing the fibrosis development, restoring the constricted lumen and decreased the submucosal connective tissue. Besides HDS, the esophageal wall thickness and the lumen diameter were measured in order to calculate the SI for all groups and. Stenosis index of the cordycepin group was comparable with those of sham group, but significantly lower than the control group, suggesting that the cordycepin produced a significant increase in the esophageal diameter and restored the normal deglutition.

In the early stages of esophageal alkali-burns, it is known that an increase in the inflammatory reaction in the connective tissue as a result of caustic burn results in increased fibroblast production, collagen production and scar formation^3^. Therefore, anti-inflammatory agents are claimed to be effective in the acute treatment of these burns. The water extract and constituents isolated from *C. militaris* were reported to be anti-inflammatory in the murine macrophage and lipopolysaccharide (LPS)/interferon (IFN)-γ stimulated macrophage cells^30^^,^ 31. The major active ingredient of *C. militaris*, cordycepin has been well documented to alleviate inflammation and oxidative stress both in vitro and in vivo^32^. Li *et al*.^32^ reported that the cordycepin administration elevated survival rate, improved liver function and suppressed hepatocyte apoptosis and necrosis in mice with severe hepatic damage. Further, they also showed that the cordycepin inhibited hepatic neutrophil and macrophage infiltration and prevented proinflammatory cytokine production including HIF-1α and TNF-α possibly through suppressing Toll like receptor 4 (TLR4) and Nuclear factor kappa B (NF-κB) signaling transduction. It was also investigated for the anti-inflammatory contribution of cordycepin in the caustic burn model by using immunohistochemical analysis and found that the cordycepin and prednisolone treatment in esophageal alkali-burn showed significant decrease in the levels of HIF-1α compared with the control group. In addition, the cordycepin treatment following caustic burn was more successful to reduce the TNF-α levels than does the prednisolone treatment, suggesting that a better anti-inflammatory effect of cordycepin in the treatment of esophageal alkali burns.

Fibroblast growth factor 2 acts to stimulate the proliferation of epithelial cells and plays a key role in the regeneration of granulation tissues. It is also demonstrated that in the acute wound, the FGF2 level is increased and plays a role in granulation tissue formation, re-epithelialization and tissue remodeling^33^. For stage II skin burns, recombinant FGF2 is applied to burns as well as skin ulcers^34^. A very recent study by Hishida *et al*.^35^ demonstrated that FGF2 treatment in a mouse burn model facilitates granulation by up-regulating the proliferation of endothelial cells and fibroblasts, suggesting to be effective in the healing process. Okata *et al*.^36^ reported that the suppression of neural damage of the esophageal wall with FGF2 in caustic esophageal injury decrease the stricture formation, which is related to the degree of innervational damage. In the present study, FGF2 protein was investigated as a marker for granulation, collagen biosynthesis and cell proliferation in the esophageal alkali-burn rats, especially in the lamina propria of esophageal mucosa. There was a higher FGF2 signal detected in the esophageal alkali-burn rats while the cordycepin and prednisolone treatment significantly reduced. However, the FGF2 was still high in the prednisolone-treated rats, suggesting that the cordycepin may be more effective in the prevention of fibrosis by interfering with the FGF2 secretion.

Vascular endothelial growth factor, an endothelial cell-specific mitogen, is the most potent angiogenic growth factor. The VEGF has been reported to response to chemokines and permeability of vascular endothelial cells. It can support the adhesion, migration and differentiation of mononuclear macrophages by inducing the formation of new blood vessels^37^. In a study by Zeytun and Özkorkmaz^38^, VEGF expression in inflammatory and endothelial cells was increased in an experimental esophageal burn rat model. In the present esophageal alkali-burn model, it was shown that high VEGF signals in small and large vascular endothelial cells were reduced by cordycepin treatment probably due to a reduction in inflammation. Here, it was predicted that the cordycepin reduces VEGF expression and vascular endothelial cell proliferation and migration, thus hindering the angiogenesis, providing less inflammation and fibrosis and promoting the healing of esophageal burns.

## Conclusions

As mentioned before, the cordycepin exhibits healing effects by reducing the granulation tissue and fibrous tissue formation and prevented the formation of esophageal strictures and exerts anti-inflammatory effects in the esophageal caustic burns. Although the duration and type of exposure (intraperitoneal/intravenous/intramuscular/oral) and dosage of cordycepin have limited the ability to achieve the broad, reproducible and detailed results, these findings suggest that the cordycepin may be used as a therapeutic agent in the acute phase of esophageal alkali-burn. Further molecular, biochemical and ultrastructural studies employing more biochemical markers will be useful in understanding the mechanism of action of cordycepin in caustic burns.
